# Is There a Role for Tranexamic Acid in Upper GI Bleeding? A Systematic Review and Meta-Analysis

**DOI:** 10.1155/2021/8876991

**Published:** 2021-01-29

**Authors:** Eoghan Burke, Patricia Harkins, Ibrahim Ahmed

**Affiliations:** ^1^Royal College of Surgeons, Dublin, Ireland; ^2^St James's Hospital, Dublin, Ireland; ^3^Our Lady of Lourdes Hospital, Drogheda, Ireland

## Abstract

**Introduction:**

Upper gastrointestinal (GI) bleeding is associated with increased morbidity and mortality. Tranexamic acid (TXA) is an antifibrinolytic agent which is licensed in the management of haemorrhage associated with trauma. It has been suggested that tranexamic acid may be able to play a role in upper GI bleeding. However, there is currently no recommendation to support this.

**Aim:**

The aim of this study was to synthesise available evidence of the effect of TXA on upper GI bleeding.

**Methods and Materials:**

A systematic review was conducted. PubMed, EMBASE, and Cochrane Central Register of Controlled Trials (CENTRAL) were searched for relevant studies. A random effects meta-analysis was performed to determine the risk ratio of primary and secondary outcomes pertaining to the use of TXA in upper GI bleeding.

**Results:**

A total of 8 studies were included in this systematic review. The total number of patients in all studies was 12994 including 4550 females (35%) and 8444 males (65%). The mean age of participants in 6 of the studies was 59.3; however the mean age for either intervention or placebo group was not reported in two of the studies. All studies reported on the effect of TXA on mortality, and the risk ratio was 0.95; however, with the 95% CI ranging from 0.80 to 1.13, this was not statistically significant. 6 of the studies reported on rebleeding rate, the risk ratio was 0.64, and with a 95% CI ranging from 0.47 to 0.86, this was statistically significant. 3 of the studies reported on the risk of adverse thromboembolic events, and the risk ratio was 0.93; however, the 95% CI extended from 0.62 to 1.39 and so was not statistically significant. 7 of the studies reported on the need for surgery, and the risk ratio was 0.59 and was statistically significant with a 95% CI ranging from 0.38 to 0.94.

**Conclusion:**

In conclusion, the use of TXA in upper GI bleeding appears to have a beneficial effect in terms of decreasing the risk of re-bleeding and decreasing the need for surgery. However, we could not find a statistically significant effect on need for blood transfusions, risk of thromboembolic events, or effect on mortality. Future randomised controlled trials may elucidate these outcomes.

## 1. Introduction

Upper gastrointestinal (GI) bleeding is defined as bleeding from the GI tract proximal to the Ligament of Treitz or the duodenojejunal flexure which delineates the transition from the duodenal to the jejunal portion of the small bowel. Acute severe GI bleeding is a common cause of death across the world [1], with upper GI bleeding being more common than lower GI bleeding. Not only is upper GI bleeding more common than lower GI bleeding, it also portends a higher mortality rate approaching 10% compared to 3% for lower GI bleeding [2]. GI bleeding is also associated with increased healthcare cost associated with interventions, need for surgery, prolonged hospital stays, and risk of rebleeding [3].

The majority of upper GI bleeding cases globally are associated with peptic ulcer disease (PUD); however, this incidence is decreasing globally secondary to the widespread eradication of *Helicobacter pylori* (*H. pylori*) with triple therapy. Other commonly encountered aetiologies include oesophagitis (infectious, inflammatory, eosinophilic, reflux), gastritis (with or without *H. pylori* as an aetiological factor), vascular abnormalities (Dieulafoy lesions, Gastric Antral Vascular Ectasia), and variceal bleeding [4].

Given the increased risk of morbidity and mortality alongside increased healthcare costs associated with managing patients presenting with acute upper GI bleeding, much research has been applied into this area in an effort to improve outcomes. Since the advent of endoscopy, and therapeutic endoscopy in particular, the mortality rates associated with acute upper GI bleeding have fallen [5]. However, there are recognized issues with access to advanced endoscopy in many resource deprived areas around the world which results in higher rates of morbidity and mortality for patients presenting with acute upper GI bleeding [6]. It is in this setting that there appears to be an increased interest in the role of tranexamic acid (TXA) as an adjunct in the management of these patients.

TXA is an antifibrinolytic agent which is licensed in the management of haemorrhage associated with trauma and major obstetric haemorrhage [7]. TXA acts by inhibiting the breakdown of fibrin by plasmin and so maintains the primary platelet plug which subsequently allows for maintenance of a thrombus [8]. It can be given via intravenous infusion or by mouth. Its use and benefit in trauma have been well studied, namely, in the CRASH 2 trial [9].

It has long been thought intuitively that TXA may be able to play a role in other instances of haemorrhage such as upper GI bleeding. However, there is currently no recommendation to support this.

The aim of this study was to synthesise available evidence of the effect of TXA on upper GI bleeding.

## 2. Materials and Methods

### 2.1. Study Aim and Objectives

The aim of this study was to synthesise available evidence of the effect of tranexamic acid on upper GI bleeding.

The primary objectives of the study are as follows:

(i)  To assess the effect of TXA versus placebo on the rate of mortality, rebleeding, and adverse events in patients with upper GI bleeding. The adverse events selected, based on literature review, focus on thrombotic adverse events, given the mechanism of action of TXA.

The secondary objectives of the study are as follows:

 (i) To assess the effect of TXA versus placebo on the need for surgery and blood product transfusion in patients presenting with upper GI bleeding.

The PICO model was used to devise the search criteria, defined in detail in Table 1.

### 2.2. Methods

#### 2.2.1. Study Design

This study was a systematic review and meta-analysis of published randomised controlled trials. Reporting of this systematic review is in accordance with the Preferred Reporting Items for Systematic Reviews and Meta-Analyses (PRISMA) statement.

#### 2.2.2. Eligibility Criteria

Inclusion criteria were as follows:

- Randomised controlled clinical trials
- Adult patients (definition depending on jurisdiction >16 or >18)
- Suspected or endoscopically verified upper GI bleeding
- Intervention: tranexamic acid, either intravenous or oral administration
- Comparator: placebo
- Outcome: must have primary outcomes reported and ideally secondary outcomes

Exclusion criteria were as follows:

  Paediatric participants.

### 2.3. Search Strategy

A detailed search strategy was developed following literature review. Key words and MeSH terms relating to tranexamic acid and upper GI bleeding were used to develop the search string: ((tranexamic acid) OR (TXA)) AND ((upper gastrointestinal bleeding) OR (upper gastrointestinal haemorrhage) OR UGIB). This search string was applied to the bibliographic databases on 22^nd^ June 2019: PubMed, EMBASE, and the Cochrane Central Register of Controlled Trials (CENTRAL). This combination of bibliographic databases was chosen based on the findings by Bramer et al. [10] on the optimum database combinations to be searched for a systematic review. We elected to use Google scholar to conduct a citation search during the study selection process, described below. This ensured that we were unlikely to miss relevant studies while limiting the amount of initial results to be screened. A variant of the above search string was applied to the relevant clinical trial registries (Clinicaltrials.gov, Japanese Medical Association Clinical Trials Registry and the EU clinical trials register) to identify potential grey literature. All databases were searched from inception.

### 2.4. Study Selection

After duplicates were removed, all of the identified studies' titles and abstracts were independently screened by two of the authors (E. Burke and P. Harkins). Abstracts meeting the previously described inclusion criteria were selected. If there was any conflict about a study's inclusion, this was resolved by a third author (I. Ahmed). The resulting studies were then reviewed in full and eligibility for inclusion in qualitative and quantitative analysis determined. Any conflict pertaining to a study's eligibility was resolved with consensus. During full article review, hand searching of references to identify any studies not identified in the original search was conducted. Similarly, a citation search using Google Scholar on all eligible articles was completed again to ensure no further studies were omitted.

### 2.5. Data Extraction

Two of the authors (E. Burke and P. Harkins) independently extracted data from the selected studies using a predetermined data extraction form. Data extracted included method of study, participant characteristics, intervention used, and outcomes including mortality, rebleeding, adverse thrombotic events, need for surgery, and transfusion requirement.

### 2.6. Risk of Bias Assessment

The quality and risk of bias in each study were assessed independently by two authors (E. Burke and P. Harkins) using the Cochrane Collaboration Risk of Bias tool [11]. This tool assesses each study's susceptibility to selection bias, performance bias, detection bias, attrition bias, and reporting bias. The susceptibility was rated as either low risk, high risk, or unclear risk. The results were depicted graphically using RevMan software.

### 2.7. Summary Measures and Synthesis of Results

We conducted a qualitative assessment (systematic review) of all eligible studies. Eligible studies reporting the outcomes were synthesised quantitatively using meta-analysis.

Studies comparing TXA versus placebo were pooled together. The relative risk for each outcome (either primary or secondary) and number of participants in each group were then extracted to facilitate a pair-wise meta-analysis to determine the risk ratio for each outcome when comparing the effect of TXA versus placebo. Risk ratios were used as the outcomes were dichotomous measures.

If at least three studies comparing TXA versus control were available, these were then compared for a given outcome (either primary or secondary). Care was taken to avoid making a unit of analysis error in the case of studies with multiple intervention or control arms.

Statistical heterogeneity amongst the studies was calculated using *I*^2^. A *P*-value of less than 0.05 was considered significant where appropriate.

The statistical analysis of the data was conducted using the Cochrane Collaboration guidelines including the use of RevMan 5.3® statistical software.

## 3. Results

### 3.1. Study Selection

**Figure 1:**
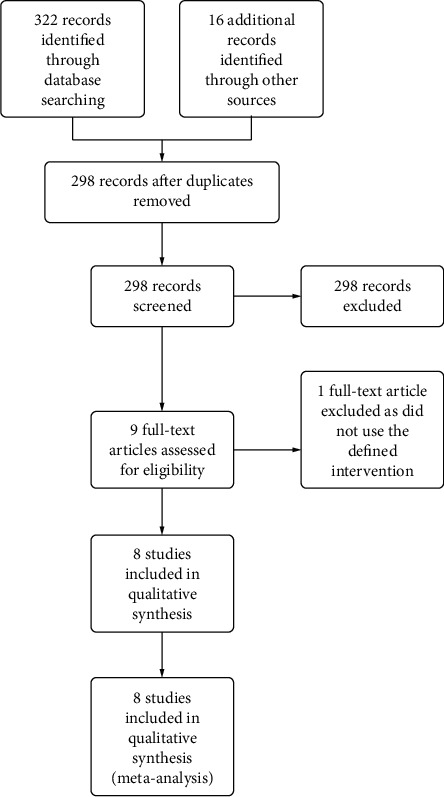
Prisma flowchart, preferred reporting items for systematic reviews and meta-analyses. This depicts the selection of studies for meta-analysis.

The number of articles found via searching the bibliographic databases PubMed, EMBASE, and Cochrane Central Register of Controlled Trials was 322. A further 16 articles were found by hand-searching of references or via citation searching on Google Scholar. Following removal of duplicates, the number of original articles to screen was 298. Screening of the title and abstract of these articles was performed independently by E. Burke and P. Harkins. Articles meeting criteria for further evaluation of full text numbered 9. Of these 9 studies, 1 was excluded as it did not use the defined intervention. Thus, 8 studies were included in the final review for narrative synthesis; all were deemed suitable to include in meta-analysis (Figure 1).

It should also be noted that the recent HALT-IT trial was included in this study even though it assessed the effects of TXA on GI bleeding in general but not isolated to upper GI sources. The reasoning behind this was the fact that the study reported that over 89% of those included in the study were deemed to have an upper GI bleed and thus it should be translatable to our study [12]. Also, this study is the largest and most recent study of its kind assessing this question and so it was deemed appropriate to include it with the above proviso acknowledged.

### 3.2. Study Characteristics

The characteristics of the studies included are detailed in Table 2. A total of 8 studies were included in this systematic review [12–19]. The number of patients included in the studies ranged from a low of 47 to a high of 12009. The total number of patients in all 8 studies was 12994 including 4550 females (35%) and 8444 males (65%). The mean age of participants in 6 of the studies was 59.3; however, the mean age for either intervention or placebo group was not reported in two of the studies, namely, Cormack 1973 and Biggs 1976.

All of the included studies used the same intervention, namely, TXA; however, there was significant heterogeneity both in terms of dose, method of administration, and duration of treatment (Table 2). All included studies used placebo as the control arm.

In terms of the reporting of both primary and secondary outcomes, all studies reported on mortality, 6 studies reported on rebleeding rates, only 3 reported on adverse thromboembolic events, 7 studies reported on the need for surgery, and 6 studies reported on transfusion requirement.

### 3.3. Risk of Bias Assessment

**Figure 2:**
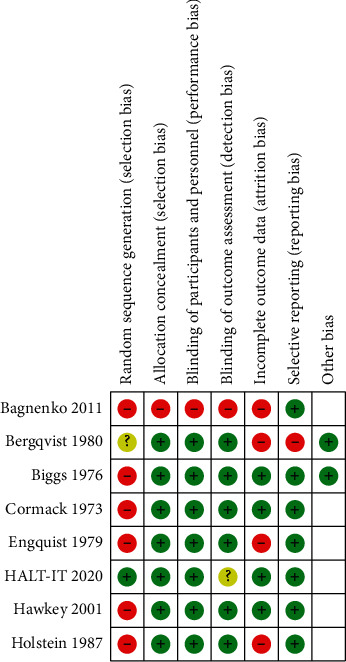
Risk of bias summary: review authors' judgements about each risk of bias item for each included study.

**Figure 3:**
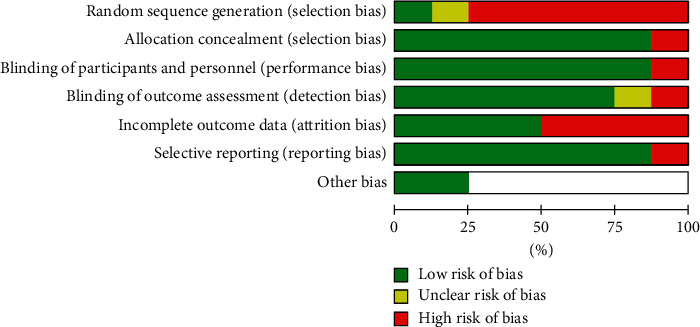
Risk of bias graph: review authors' judgements about each risk of bias item presented as percentages across all included studies.

Study quality in terms of risk of bias was assessed independently by E. Burke and P. Harkins using the Cochrane Collaboration Risk of Bias tool. The results are outlined in Figures 2 and 3.

### 3.4. Synthesis of Results for Meta-Analysis

A random effects meta-analysis was then performed to determine the risk ratio of a given outcome comparing the TXA group to the placebo group.

**Figure 4:**
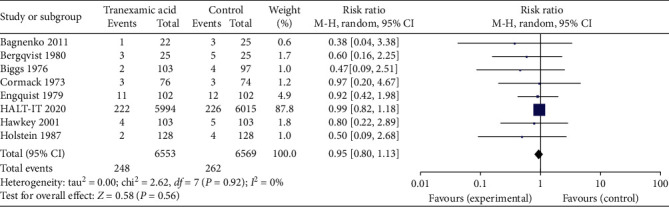
Forest plot of effect of tranexamic acid versus placebo on mortality.

All 8 studies reported on mortality as an outcome. Results were graphed in a forest plot (Figure 4). This revealed a risk ratio of 0.95 with a 95% confidence interval (CI) of between 0.80 and 1.13. The *Z* statistic for the overall effect size was 0.58 but was not statistically significant with a *P*-value of 0.56. The included studies were homogenous as evidenced by the *I*^2^ value of 0%.

#### 3.4.1. Rebleeding Rate

**Figure 5:**
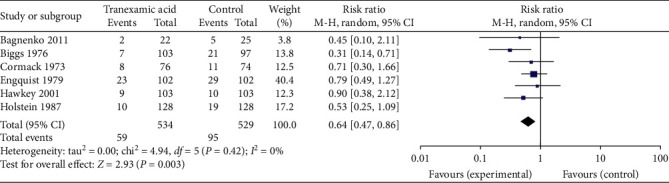
Forest plot of effect of tranexamic acid versus placebo on rebleeding rate.

6 of the studies reported on rebleeding rate as an outcome. Results were graphed in a forest plot (Figure 5). This revealed a risk ratio of 0.64 with a 95% CI of between 0.47 and 0.86. The *Z* statistic for the overall effect size was 2.93 and was statistically significant with a *P*-value of 0.003. The included studies were homogenous as evidenced by the *I*^2^ value of 0%.

#### 3.4.2. Adverse Thromboembolic Events

**Figure 6:**
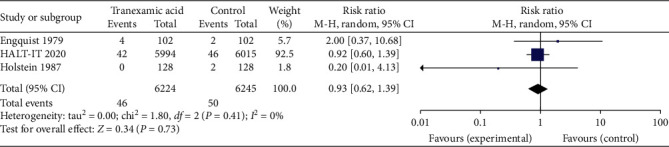
Forest plot of effect of tranexamic acid versus placebo on risk of thrombotic events, namely, myocardial infarction, cerebrovascular accident, and pulmonary embolism.

3 of the studies reported on adverse thromboembolic events (namely, myocardial infarction, cerebrovascular accidents, and pulmonary emboli). Results were graphed in a forest plot (Figure 6). This revealed a risk ratio of 0.93 with a 95% CI of between 0.62 and 1.39. The *Z* statistic for the overall effect size was 0.34 but was not statistically significant with a *P* value of 0.73. The included studies were homogenous as evidenced by the *I*^2^ value of 0%.

#### 3.4.3. Need for Surgery

**Figure 7:**
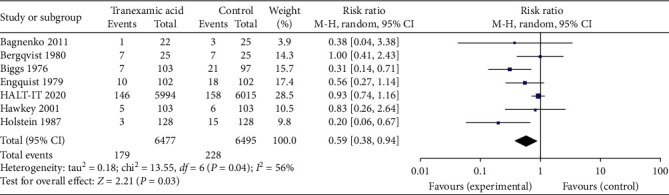
Forest plot of effect of tranexamic acid versus placebo on need for surgery.

7 of the studies reported on the subsequent need for surgery in these patients enrolled in the study. Results were graphed in a forest plot (Figure 7). This revealed a risk ratio of 0.59 with a 95% CI of between 0.38 and 0.94. The *Z* statistic for the overall effect size was 2.21 and was statistically significant with a *P*-value of 0.03. The included studies were heterogenous as evidenced by the *I*^2^ value of 56%.

#### 3.4.4. Need for Transfusion

**Figure 8:**
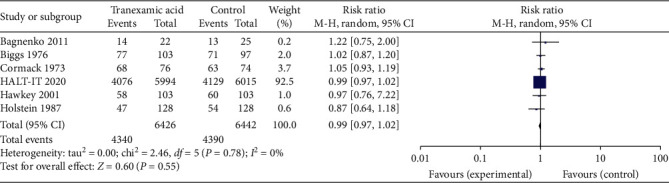
Forest plot of effect of tranexamic acid versus placebo on need for transfusion.

6 of the studies reported on transfusion requirement. Results were graphed in a forest plot (Figure 8). This revealed a risk ratio of 0.99 with a 95% CI of between 0.97 and 1.02. The *Z* statistic for the overall effect size was 0.60 and was not statistically significant with a *P*-value of 0.55. The included studies were homogenous as evidenced by the *I*^2^ value of 0%.

## 4. Discussion

TXA was discovered in Japan in 1962 by two Japanese researchers, Shosuke and Utako [20]. Their research had focused on the inhibition of enzymatic breakdown of fibrin. The chemical they initially discovered, which displayed this ability, was AMCHA later referred to as tranexamic acid (TXA) [21]. TXA is a synthetic derivative of the amino acid lysine that inhibits fibrinolysis by blocking the interaction of plasminogen with the lysine residues of fibrin [22].

Initially, TXA was marketed as an agent which could be used in the management of mild bleeding associated with dental procedures. However in 1966, again in Japan, Kobayashi and Sugiura published a paper in which they examined the role of tranexamic acid in obstetric haemorrhage [23]. Their results were impressive indicating a significant reduction in blood loss, need for transfusion, and risk of mortality. Following this seminal study, multiple studies were then conducted assessing the role of this antifibrinolytic agent in multiple different fields. Most contemporaneously, the CRASH 2 trial examined the role of TXA in trauma [24].

To date, the role of TXA in upper GI bleeding is not routine practice. Our extensive literature search identified 8 relevant randomised controlled trials which examined the effect of TXA on multiple outcomes pertaining to upper GI bleeding. Our narrative review of these studies revealed significant heterogeneity amongst the studies in relation to mode of administration, dose of TXA, and duration of treatment. This systematic review and meta-analysis is the first to incorporate the data from the large HALT-IT trial. However unfortunately this trial did not focus specifically on upper GI bleeding nor did it assess some of the primary and secondary outcomes of interest.

Our study has found that the use of TXA in upper GI bleeding decreases the rebleeding rate with a risk ratio of 0.64 favouring the TXA group and with a 95% CI ranging from 0.47 to 0.86; this was statistically significant. We can also say that the use of TXA in this setting decreases the need for surgery in this cohort of patients presenting with upper GI bleeding with a risk ratio of 0.59 favouring the TXA group and a 95% CI ranging from 0.38 to 0.94 confirming statistical significance.

However, our results in relation to the remaining primary and secondary outcomes were less clear. All 8 studies reported on the effect of TXA on mortality; however, as the 95% CI ranged from 0.80 to 1.13, its effect is not statistically significant. Similarly, in the 3 studies which reported on the risk of adverse thromboembolic events the 95% CI extended from 0.62 to 1.39 and so, again, was not statistically significant. Furthermore, when we consider the 6 studies that assessed the effect of TXA on the need for transfusion in upper GI bleeding, the risk ratio of 0.99 had a 95% CI ranging from 0.97 to 1.02 and so was not statistically significant.

Currently, there are a further two studies assessing the role of TXA in upper GI bleeding. The efficacy and tolerance of early administration of TXA in patients with cirrhosis presenting with acute upper gastrointestinal bleeding study (the EXARHOSE study) is currently enrolling [25] whilst the Tranexamic Acid for Upper Gastrointestinal Bleeding (TAUGIB) study in South Korea has recruited 414 patients to date [26]. The combination of results from both the TAUGIB and EXARHOSE studies will indeed by informative in future use of TXA in upper GI bleeding. These studies will hopefully shed more light on the effect of TXA on mortality, risk of adverse thromboembolic events, and need for transfusion.

The limitations of this present systematic review and meta-analysis include the heterogeneity of the included studies. The heterogeneity pertains to sample size, dose of TXA used, and duration of treatment. There was also a degree of heterogeneity amongst the included studies in relation to reporting of the primary and secondary outcomes of this review.

## 5. Conclusions

TXA use in upper GI bleeding does appear to decrease the risk of rebleeding and decrease the need for surgical intervention. However, its effect on mortality, adverse events, and transfusion requirements remains unclear. Future randomised controlled trials may elucidate these outcomes.

## Figures and Tables

**Table 1:** PICO model used to define search criteria for search strategy to be used in the relevant bibliographic databases.

*P*	*I*	*C*	*O*
Population	Intervention	Comparison	Outcome
Adult patients presenting with upper GI bleeding	Tranexamic acid given intravenously or by mouth	Placebo	Primary and secondary outcomes as outlined above

**Table 2:** Summary of studies included in systematic review.

Study ID	Characteristics
HALT-IT 2020	*Methods*:
	Randomised controlled trial
	*Participants*:
	12009 patients randomly assigned
	Inclusion criteria:
	GI bleed (upper or lower, however 89% were classified as upper GI bleeds)
	Mean age TXA group: 58
	Mean age placebo group: 58
	7743 males
	4266 females
	*Interventions*:
	TXA 1 g IV stat then 3 g infused over 24 hours
	*Outcomes*:
	Mortality:
	TXA group 222/5994
	Placebo group 226/6015
	Rebleeding:
	Not reported
	Adverse events MI, CVA, PE:
	TXA group 42/5994
	Placebo group 46/6015
	Need for surgery:
	TXA group 146/5994
	Placebo group 158/6015
	Transfusion required:
	TXA group 4076/5994
	Placebo group 4129/6015
Bagnenko 2011	*Methods*:
	Randomised controlled trial
	*Participants*:
	47 patients randomly assigned
	Inclusion criteria:
	Suspected upper GI bleed
	Mean age TXA group: 62
	Mean age placebo group: 64
	29 males
	18 females
	*Interventions*:
	TXA 10 mg IV/PO TDS for 3 days versus placebo
	*Outcomes*:
	Mortality:
	TXA group 1/22
	Placebo group 3/25
	Rebleeding:
	TXA group 2/22
	Placebo group: 5/25
	Adverse events:
	Not reported
	Need for surgery:
	TXA 1/22
	Placebo 3/25
	Transfusion required:
	TXA 14/22
	Placebo 13/25
Hawkey 2001	*Methods*:
	Randomised controlled trial
	*Participants*:
	206 patients randomly assigned.
	Inclusion criteria:
	Suspected upper GI bleed
	Mean age TXA group: 58
	Mean age placebo group: 58
	126 males
	80 females
	*Interventions*:
	TXA 2 g PO bolus then 1 g QDS for 4 days
	*Outcomes*:
	Mortality:
	TXA group 4/103
	Placebo group 5/103
	Rebleeding:
	TXA group 9/103
	Placebo group: 10/103
	Adverse events:
	No breakdown between intervention and control
	Need for surgery:
	TXA 5/103
	Placebo 6/103
	Transfusion required:
	TXA 58/103
	Placebo 60/103
Holstein 1987	*Methods*:
	Randomised controlled trial
	*Participants*:
	128 patients randomly assigned
	Inclusion criteria:
	Suspected upper GI bleed
	Mean age TXA group: 62
	Mean age placebo group: 65
	90 males
	38 females
	*Interventions*:
	TXA 1 g every 4 hours for 24 hours then 1.5 g PO QDS for 5 days
	*Outcomes*:
	Mortality:
	TXA group 2/128
	Placebo group 4/128
	Rebleeding:
	TXA group 10/128
	Placebo group 19/128
	Adverse events MI, CVA, PE:
	TXA group 0/128
	Placebo group 2/128
	Need for surgery:
	TXA group 3/128
	Placebo group 15/128
	Transfusion required:
	TXA group 47/128
	Placebo group 54/128
Bergqvist 1980	*Methods*:
	Randomised controlled trial
	*Participants*:
	50 patients randomly assigned
	Inclusion criteria:
	Suspected upper GI bleed
	Mean age TXA group: 61
	Mean age placebo group: 58
	40 males
	10 females
	*Interventions*:
	TXA 2 g PO 4 hourly for two days
	*Outcomes*:
	Mortality:
	TXA group 3/25
	Placebo group 5/25
	Rebleeding:
	Not reported
	Adverse events MI, CVA, PE:
	Not reported
	Need for surgery:
	TXA group 7/25
	Placebo group 7/25
	Transfusion required:
	Not reported

Engquist 1979	*Methods*:
	Randomised controlled trial
	*Participants*:
	204 patients randomly assigned
	Inclusion criteria:
	Suspected upper GI bleed
	Mean age TXA group: 59
	Mean age placebo group: 56
	159 males
	45 females
	*Interventions*:
	TXA 1 g IV 4 hourly for 1 day then 1.5 g PO QDS for 6 days
	*Outcomes*:
	Mortality:
	TXA group 11/102
	Placebo group 12/102
	Rebleeding:
	TXA group 23/102
	Placebo group 29/102
	Adverse events MI, CVA, PE:
	TXA group 4/102
	Placebo group 2/102
	Need for surgery:
	TXA group 10/102
	Placebo group 18/102
	Transfusion required:
	Not reported
Biggs 1976	*Methods*:
	Randomised controlled trial
	*Participants*:
	200 patients randomly assigned
	Inclusion criteria:
	Suspected upper GI bleed
	Mean age TXA group: not reported
	Mean age placebo group: not reported
	156 males
	44 females
	*Interventions*:
	TXA 1 g IV stat then 1 g PO QDS on day 1,
	Then 1 g QDS for 4 days
	*Outcomes*:
	Mortality:
	TXA group 2/103
	Placebo group 4/97
	Rebleeding:
	TXA group 7/103
	Placebo group 21/97
	Adverse events MI, CVA, PE:
	Not reported
	Need for surgery:
	TXA group 7/103
	Placebo group 21/97
	Transfusion required:
	TXA group 77/103
	Placebo group 71/97

Cormack 1973	*Methods*:
	Randomised controlled trial
	*Participants*:
	150 patients randomly assigned
	Inclusion criteria:
	Suspected upper GI bleed
	Mean age TXA group: not reported
	Mean age placebo group: not reported
	101 males
	49 females
	*Interventions*:
	TXA 1.5 g QDS for 7 days
	*Outcomes*:
	Mortality:
	TXA group 3/76
	Placebo group 3/74
	Rebleeding:
	TXA group 8/76
	Placebo group 11/74
	Adverse events MI, CVA, PE:
	Not reported
	Need for surgery:
	Not reported
	Transfusion required:
	TXA group 68/76
	Placebo group 63/74

## Data Availability

The data are freely available via search strategy as this is a systematic review and meta-analysis of published articles.
